# Eye-tracking analyses of physician face gaze patterns in consultations

**DOI:** 10.1038/s41598-021-99068-4

**Published:** 2021-10-06

**Authors:** C. Jongerius, H. G. van den Boorn, T. Callemein, N. T. Boeske, J. A. Romijn, E. M. A. Smets, M. A. Hillen

**Affiliations:** 1 Department of Medical Psychology, Amsterdam Public Health, Amsterdam UMC, Location AMC, University of Amsterdam, Meibergdreef 9, 1100 DD Amsterdam, The Netherlands; 2 PSI-EAVISE, Electrical Engineering Technology (ESAT), KU Leuven, De Nayer Campus Sint-Katelijne-Waver, Sint-Katelijne-Waver, Belgium; 3 Department of Medicine, Amsterdam UMC, University of Amsterdam, Amsterdam, The Netherlands; 4 Department of Medical Oncology, Amsterdam UMC, Location AMC, University of Amsterdam, Amsterdam, The Netherlands

**Keywords:** Psychology, Human behaviour

## Abstract

Face gaze is a fundamental non-verbal behaviour and can be assessed using eye-tracking glasses. Methodological guidelines are lacking on which measure to use to determine face gaze. To evaluate face gaze patterns we compared three measures: duration, frequency and dwell time. Furthermore, state of the art face gaze analysis requires time and manual effort. We tested if face gaze patterns in the first 30, 60 and 120 s predict face gaze patterns in the remaining interaction. We performed secondary analyses of mobile eye-tracking data of 16 internal medicine physicians in consultation with 100 of their patients. Duration and frequency of face gaze were unrelated. The lack of association between duration and frequency suggests that research may yield different results depending on which measure of face gaze is used. Dwell time correlates both duration and frequency. Face gaze during the first seconds of the consultations predicted face gaze patterns of the remaining consultation time (R^2^ 0.26 to 0.73). Therefore, face gaze during the first minutes of the consultations can be used to predict face gaze patterns over the complete interaction. Researchers interested to study face gaze may use these findings to make optimal methodological choices.

## Introduction

Face gaze is a fundamental aspect of nonverbal behaviour. Face gaze assessed with eye-tracking glasses can be studied using three measures: duration, frequency and dwell time. There is no evidence that shows how the three measures are related or whether one of these measures yields a more meaningful indication of face gaze. Furthermore, for the sake of researchers’ analyses, knowing whether an initial slice of face gaze data is associated to face gaze during the remaining interaction would be worthwhile studying to reduce the analytic burden associated with large datasets. Therefore, we want to test which face gaze measure yields more meaning and whether slices of face gaze are indicative of face gaze during a complete interaction.

Face gaze is crucial for transmitting social, emotional and attentional information^1–3^. Hence, the understanding of face gaze is essential for the interpretation of nonverbal communication and its effects on interpersonal relationships. Face gaze has been studied in various scientific disciplines, including communication sciences, social psychology and psychiatry^4^. Face gaze is often used as a proxy for eye gaze^4,5^. Numerous findings show that the perception of eye gaze (or eye contact) is an overestimation of the perceiver based on face gaze^5–8^. Therefore, face gaze may be an indication of the perceiver’s subjective eye contact experience.

In previous studies, a variety of methods has been used to assess face gaze^4^. The generalizability and comparability of these studies is complex. Examples of methods are the ranking of face gaze by human assessors on a coding sheet, or the evaluation of the level of face (or eye) gaze based on video registrations^9,10^. The most commonly used technique is assessment using a video camera^4^. A camera films the interactors and the level of face gaze is derived from these video registrations, which may involve timing the duration of face gaze by pressing a button every time the coder observes that face gaze takes place^11,12^. Although assessment of the level of face gaze by coders has been used for decades and has been extensively tested, this method is labour intensive and by definition vulnerable to coder subjectivity, and moreover to a lack of accuracy^13^.

These methodological limitations of manual video coding can be overcome using mobile eye-tracking^5,6,14–16^. Eye-tracking glasses have a scene camera that records the world view in front of the participant^17^. This scene camera is positioned on the nose bridge. Simultaneously, infrared cameras integrated in the glasses film the eyes of the person wearing the glasses^17^. The software developed along with these wearable eye-tracking glasses uses data from both cameras to calculate the focus of gaze^17^. The output of mobile eye-tracking registrations is a video of the wearer’s head perspective with information about their gaze focus, often depicted as a dot on the video image (i.e., a 2D position (pixel) on a video screen). Specific software can be used to automatically assess the gaze location in the scene camera (e.g., whether the gaze is focused within a pre-specified region of interest), thus reducing subjectivity in coding and enhancing precision^18^. Wearable eye-tracking techniques moreover enable studying an individual’s gaze and the effects thereof more quickly and reliably compared to conventional techniques.

When using wearable eye-tracking technologies to assess face gaze, several methodological choices need to be made. At least two of these choices are essential for the interpretation of eye tracking data. First, comparing results between studies assessing face gaze may be facilitated if studies use similar measures. It is important to understand which measure is most appropriate when assessing face gaze. Second, analysing mobile eye tracking data may be a time intensive process^18^. Data collection in routine settings, e.g. clinical consultations, may be more prone to error and invasive than data collection in experimental settings. Therefore, methods to reduce the time required for measuring or analysing face gaze could be helpful to improve the feasibility of studying face gaze across disciplines.

A first crucial methodological issue when analysing mobile eye-tracking videos of real life interactions is how to measure face gaze. Three measures have been frequently used: duration, frequency and dwell time of face gaze. Duration of face gaze is assessed by calculating the absolute or relative duration of face gaze within an interaction (i.e., gaze time)^4,19^. Duration of face gaze is how long the gaze is directed towards the face. Frequency of face gaze represents the number of times in total (absolute) or per unit of time (relative) in which face gaze takes place within an interaction (i.e., gaze count)^4,19^. Frequency of face gaze is a change in direction of gaze from an undefined area towards the face. A third measure, dwell time, indicates the duration per instance of gaze within a specific Area-of-Interest^18^. Dwell time of face gaze is the duration of one instance of uninterrupted gaze towards the face.

Previous research indicates that either a long duration or a high frequency of eye/face gaze are preferable in interaction^20,21^. For example, low levels of gaze between physicians and patients were suggested to have negative effects on physician–patient relationships^22–24^. Accordingly, short duration and low frequency of face/ eye gaze have been suggested to indicate impaired face gaze, which in turn has been associated with psychopathology^1,16,25–28^. However, to the best of our knowledge there is no evidence that shows how the three measures, i.e., duration, frequency and dwell time, are related to each other, nor is it known whether one of these measures yields a more meaningful indication of face gaze.

A second crucial methodological issue when analysing mobile eye-tracking videos of real life interactions is that it generates a large amount of data. Eye tracking provides researchers with a gaze location for each several milliseconds. So far, studies using wearable eye-tracking glasses during face-to-face interactions have used relatively small sample sizes, possibly because of the large amount of data that are generated^7,14,16,29–31^. Previous research has shown that analysing vast amounts of wearable eye-tracking data can be challenging, especially in applied settings^32^. A possible solution to this problem is to use thin-slicing, which involves making inferences about behaviour based on narrow windows of time and information^33^. State of the art algorithms and analysis techniques for studying face gaze still require a considerable amount manual work and time^34^. It can therefore be helpful to use brief segments of data, if the same conclusions can be made using thin slices. Research using thin slicing to study gaze (without eye-tracking techniques) has used the first segment (range 5–60 s) of a conversation to make inferences about the whole interaction^35–37^. These studies compared the conclusions based on thin slices of face gaze to those based on the remaining duration of the conversation and found that the use of thin slices was valid and reliable ^36^. These findings should be validated using eye-tracking. Moreover, it appears that gaze during the first phase of a conversation, such as during the first instances of a patient-physician consultation, is highly consequential for the structure of the interaction^38^. It is presently unclear, however, whether face gaze assessed by wearable eye-tracking glasses during the first short fragment of an interaction is predictive of face gaze during the entire interaction.

We aimed to answer two research questions. First, what is the relation between three measures of face gaze, i.e. duration, frequency and dwell time, in real life interactions? Second, is face gaze during the brief first period of the consultation, i.e., 30 s, 1 min or 2 min, a reliable predictor for face gaze during the remainder of the consultation, for all three measures? We test our research questions using an existing eye-tracking dataset of physician gaze while consulting patients in an internal medicine out-patient clinic.

## Results

### Descriptive characteristics

#### Sample characteristics

In total, 16 physicians participated in this study. Those physicians conducted consultations with a total of 100 participating patients. Physicians saw between 2 and 14 patients (median = 6). Sociodemographic characteristics of physicians and patients are specified in Table 1.

**Table 1:** Sociodemographic characteristics of physicians and patients.

Physicians (N = 16)	Mean (range/percentage)	SE
Age in years	33.9 (29–38)	0.6
Female gender	8 (50%)	
**Self-identified nationality**		
Dutch	15 (94%)	
Arabic	1 (6%)	

Patients (N = 100)	Mean (range/percentage)	SD
Age	58.1 (25–82)	14.0
Female gender	47 (47%)	
**Self-identified nationality**		
Dutch	94 (94 %)	
Other European nationalities	2 (2%)	
South American	3 (3%)	
Middle East	1 (1%)	
**Educational level (n = 99)**		
None/primary school	29 (29%)	
Secondary/lower level vocational school	50 (50%)	
Higher vocational training/university	20 (20%)	

#### Consultation characteristics

The consultations concerned various diseases, resulting in large variation regarding the discussed topics, ranging from medically unexplained symptoms, to cancer, to diabetes and cardiovascular diseases. Physical examination was performed in 24 of the consultations (24%) and an informal caregiver (i.e., spouse, other family member, or friend) was present in 17 consultations (17%). The median consultation duration (disregarding the physical examinations) was 14.1 min (ranging from 3.0 to 45.2 min).

### Characteristics of face gaze measures

#### Face gaze duration

**Figure 1:**
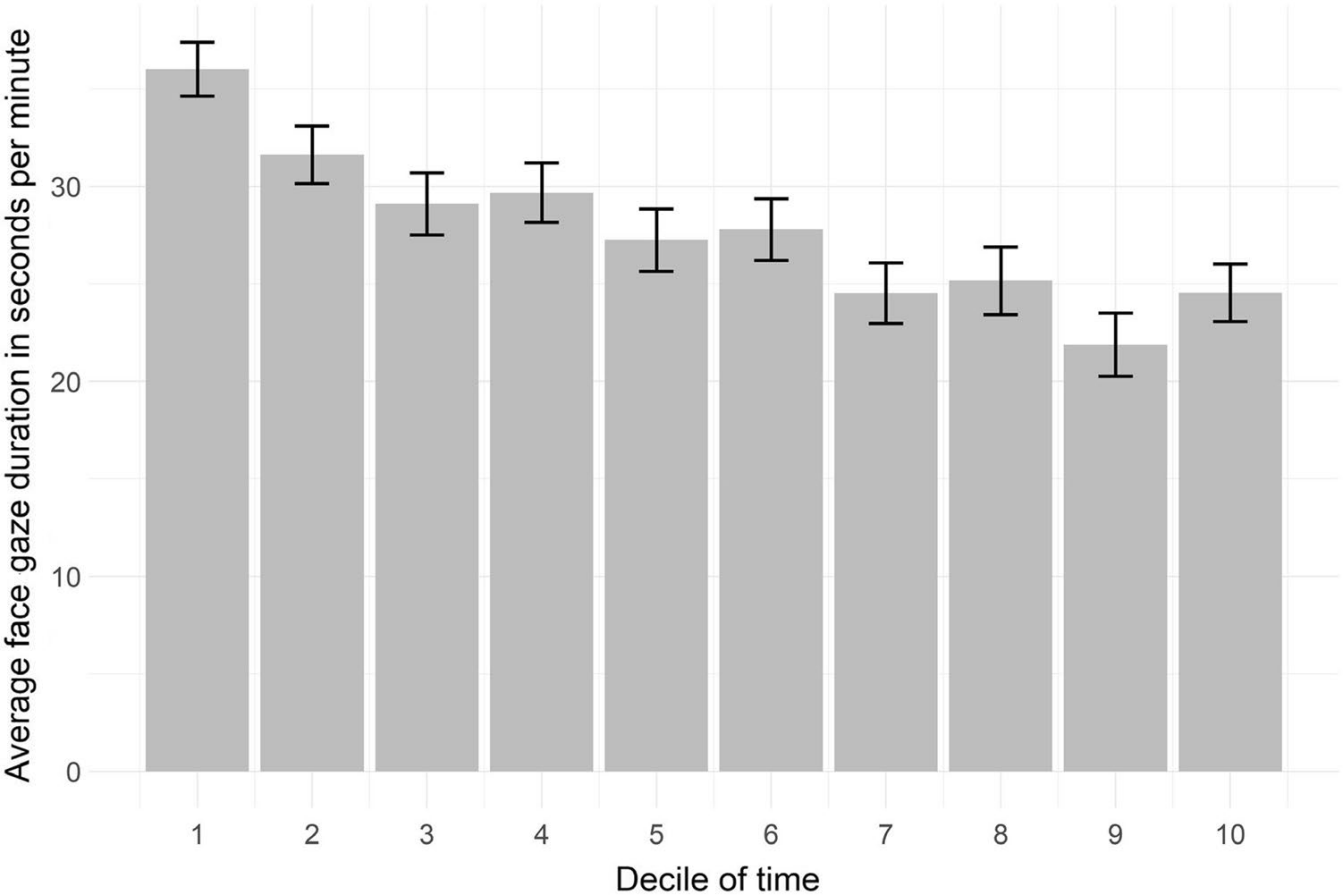
Development of the physicians’ face gaze duration per minute throughout the consultations per decile of time.

The median duration of face gaze from physician to patient was 27.8 s per minute (range 6.0–49.9). On average physicians gazed at the patient’s face 48% of the consultation time. The average face gaze duration per minute for every decile of time illustrates that relative face gaze duration decreased throughout the consultation (Fig. 1) The longest average face gaze occurred in the first decile (36.0 s, SE = 1.4 s) and the shortest in the ninth decile (21.9 s, SE = 1.6 s). The time-series analysis confirmed an overall slight but significant decrease (β = − 1.26, *P* < 0.001). Face gaze duration per minute was not correlated to patient satisfaction (r = 0.039, *P* = *0.6*99). Furthermore, the longer the consultation lasted, the greater the decrease in average face gaze duration per minute (r = − 0.25, *P* = 0.01).

#### Face gaze frequency

**Figure 2:**
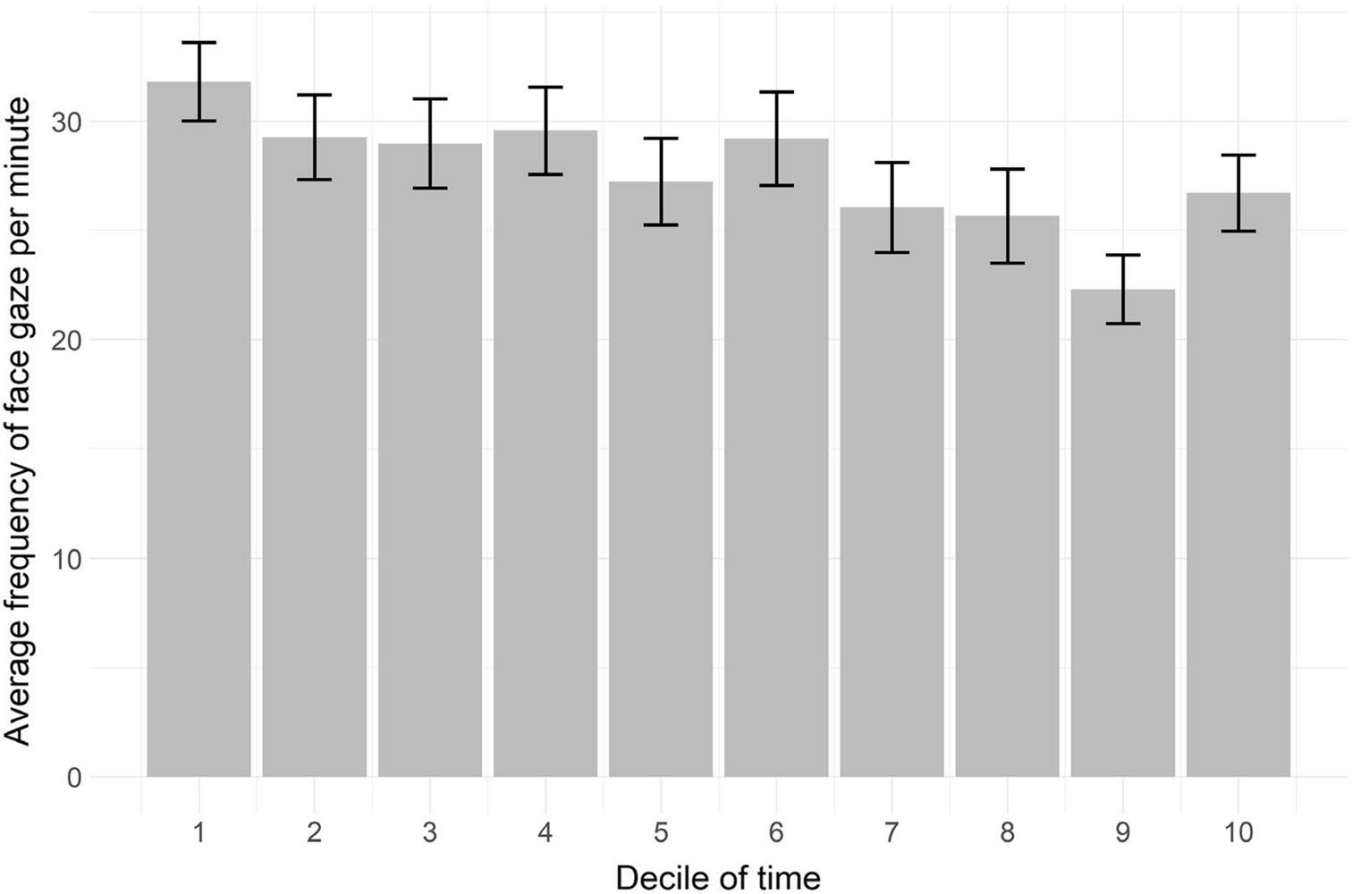
Development of the physicians’ face gaze frequency per minute throughout the consultations per decile of time.

The median frequency of face gaze from physician to patient was 25 times per minute (range 7–76). The face gaze frequency per minute for every decile of time illustrates a decrease over the course of the consultation (Fig. 2). Frequency was highest in the first decile (31.8 times per minute, SE = 1.8 times per minute) and lowest in the ninth decile (22.3 times per minute, SE = 1.6 times per minute). The time-series analysis indicated that this overall decrease was small but significant (β = − 0.76, *P* < 0.001). Frequency of face gaze per minute was positively and significantly related to patient satisfaction (r = 0.309, *P* = 0.002), but not to the consultation duration(r = − 0.045, *P* = 0.653).

#### Face gaze dwell time (per instance)

**Figure 3:**
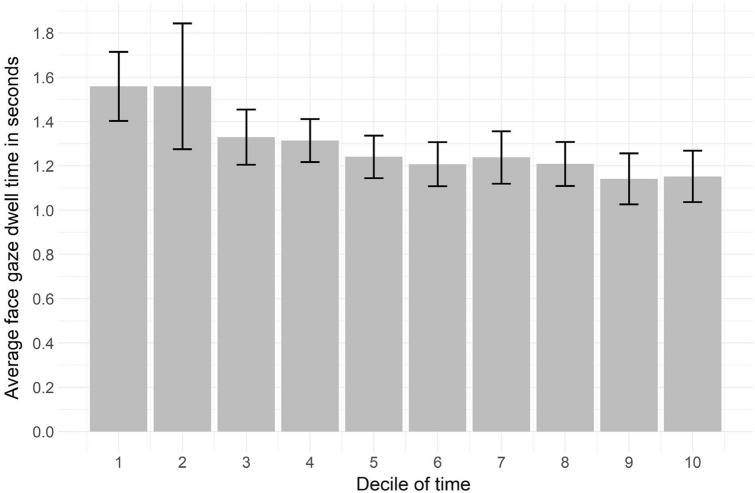
Development of the physicians’ face gaze dwell time in seconds throughout the consultations per decile of time.

Physicians’ median dwell time on the patients’ face (the median duration per instance of face gaze) was 1.0 s (ranging from the minimal dwell time of 0.1 to 6.8 s). Figure 3 shows the average face gaze dwell time in seconds for every decile of the total consultation time. The longest average dwell time occurred in the second decile (1.5 s, SE = 0.3 s) and the shortest in the ninth decile (1.1 s, SE = 0.1 s). On average the face gaze dwell time decreased during the consultation. The time series analysis indicated a slight but significant overall decrease (β = − 0.036, *P* = 0.013). The average dwell time was not related to patient satisfaction (r = − 0.184, *P* = 0.066) or consultation duration (r = − 0.190, *P* = 0.058).

### Research question 1: correlations between different measures of face gaze

Face gaze duration per minute and face gaze frequency per minute were not correlated (*r* = 0.16, *P* = 0.12). A significant positive relation was found between face gaze duration per minute and face gaze dwell time (*r* = 0.51, *P* < 0.001). Face gaze frequency and face gaze dwell time correlated negatively and significantly (*r* = − 0.54, *P* < 0.001).

### Research question 2: multilevel testing of the association between the first brief period of face gaze and face gaze during the remainder of the consultation

Table 2 displays the results of the linear models testing the association between the first 30, 60 and 120 s of face gaze and face gaze during the remainder of the consultation separately for all three measures (duration per minute, frequency per minute and dwell time). For all three measures, face gaze during the first slices (30, 60 and 120 s) was positively and significantly associated with the face gaze in the remainder of the consultation (see Table 2). The strongest associations were found when comparing the first 120 s with the remaining face gaze for all face gaze measures (the R^2^ were 39% for duration, 37% for frequency, and 73% for dwell time). Overall, the larger the slice, the stronger the association: the R-squared increased when the duration of the slice increased, with the biggest increases from 30 to 60 s. In most models consultation duration did not affect strength of the association between face gaze in the 30, 60, 120 slices and the remainder of the consultation. Two exceptions were the first 30 and 60 s of face gaze duration per minute. In the first 30 and 60 s the duration of the consultation significantly contributed to the outcome of the models. This means that consultation duration affected the strength of the association between the first 30 and 60 s of face gaze duration and the face gaze duration in the remaining part of the consultation. Because each physician consulted with multiple patients, we tested the same models adding a random effect per physician, expecting that accounting for within-physician variation would improve our models. However, adding this random effect did not improve model fit indicating that there were no consistent patterns in face gaze within physicians (data shown in Supplementary Table S1).

**Table 2:** Results of the linear regression models to predict face gaze duration per minute, frequency per minute, and dwell time with a varying slice time of 30, 60 or 120 s.

Face gaze outcome	Linear regression models				
Face gaze duration in seconds per minute	Duration of the predicting slice	Intercept	Face gaze duration in seconds in the predicting slice	Total duration of the consultation in minutes	R^2^
	30 s	31.5***	0.74***	− 0.33**	0.26%
	60 s	20.6***	0.52***	− 0.30**	0.39%
	120 s	18.9***	0.27***	− 0.21 (NS)	0.39%

Face gaze frequency per minute	Duration of the predicting slice	Intercept	Face gaze frequency in the predicting slice	Total duration of the consultation in minutes	R^2^
	30 s	18.0***	0.69***	− 0.22 (NS)	0.28%
	60 s	15.3***	0.44***	− 0.22 (NS)	0.36%
	120 s	10.3**	0.27***	− 0.03 (NS)	0.37%

Face gaze dwell time in seconds	Duration of the predicting slice	Intercept	Face gaze dwell time in seconds in the predicting slice	Total duration of the consultation in minutes	R^2^
	30 s	0.85***	0.43***	− 0.01 (NS)	0.54%
	60 s	0.77***	0.38***	− 0.01 (NS)	0.66%
	120 s	0.68***	0.45***	− 0.01 (NS)	0.73%

NS: not significant, *p < 0.05, **p < 0.01, ***p < 0.001.

For each outcome, we used the level of face gaze in the initial slice, as well as total consultation duration in minutes and a model intercept. The model parameters are displayed for each linear model as well as the model R-squared.

## Discussion

We tested the use of three different measures of face gaze—i.e., duration, frequency and dwell time—in real life physician–patient outpatient interactions. Our results show that the two measures duration and frequency of face gaze are unrelated. This means that the choice of one versus the other of these measures will lead to different findings and consequently possibly to different conclusions. Dwell time, i.e., the duration per instance of gaze, is calculated using duration and frequency of face gaze, and the resulting value therefore relates to both measures. Furthermore, we found strong associations between face gaze during the first 30, 60 and 120 s of interactions and face gaze during the remainder of the interaction for all three face gaze measures. In particular, dwell time has the highest associations between the first instances of face gaze and the remaining face gaze. From a methodological perspective, this implies that the first instances of face gaze can be used to draw global conclusions about face gaze over a whole interaction, especially when using dwell time.

Our finding that duration and the frequency of face gaze were unrelated measures, suggests that both measures assess different aspects of face gaze. Therefore, comparisons between the results of studies that use either duration or frequency of face gaze should be made cautiously. Both duration and frequency are captured in the dwell time measure. Dwell time, which is the duration per instance of gaze, is calculated using duration and frequency of face gaze. The measures are different in terms of content. A long duration can mean that a person is staring, a high frequency can mean that a person is looking back and forth restlessly, a long dwell time captures both the durantion and the frequency of gazing. Therefore, dwell time may be the most informative measure of face gaze. Future face gaze analyses may include at least dwell time, possibly complemented with either frequency or duration of face gaze.

Interestingly, our time series analyses illustrate that duration, frequency and dwell time follow a similar pattern over the course of these medical interactions, i.e., longer and higher instances in the start, decreasing towards the end of the consultation. Duration and frequency did not correlate, but do follow a similar face gaze pattern. The higher amount of face gaze at the beginning of the consultation may be due to the unfamiliarity of the interactants^39^. Furthemore, the decrease in face gaze may be due to the structured nature of medical follow-up consultations. This interaction starts with greeting (initiating the session), followed by information gathering, a possible physical examination, and finally the end of the consultation^40^. Different patterns may be observed in other types of interactions and it would be worthwhile to investigate whether there are similar patterns in interactions of different settings.

Wearable eye-tracking glasses enable new opportunities and specificity to measure face gaze. We used wearable eye-tracking glasses to study physicians’ gaze towards patients’ faces in regular follow-up consultations. Our finding that the first 30, 60 and 120 s of face gaze strongly predict face gaze in the remainder of the interaction suggests that individuals may have consistent personal styles in their gaze behaviour. However, contrary to previous research, in our study we did not find patterns in the face gaze behavior of individual physicians across their interactions, possibly because earlier results focussed on gaze behaviour differences within the face area^5,15^. Therefore, we attribute the association between the first instance and the remainder of face gaze to the interaction between two specific individuals, in which each interactor brings unique element to the face gaze behavior, and consequently the interaction. This matches earlier findings, showing that face-to-face communication is the product of the interaction of two individuals’ gaze behaviour^39^. This latter study, involving dual eye-tracking, indicated that mutual gaze correlated with the combined characteristics of the interactants such as whether the interactors were familiar with each other. Gaze in interaction should therefore be considered as an outcome of the characteristics of the combination of interactors rather than those of one interactor in isolation^39^.

The large variation of physician face gaze patterns and its influence on patient satisfaction deserve further investigation^5^. We found that higher face gaze frequency, but not duration or dwell time, was associated with more patient satisfaction. A possible explanation for the large variation and the influence on satisfaction could be the role of gaze in directing the conversation^41^. One experimental study shows that gaze was especially useful in turn taking management, i.e., signalling who could be the next speaker^41^. The previous study showed that gaze was used to organize speech between the speaker and the listener^41^. Therefore, a higher frequency could indicate a more interactive conversation, i.e., more turn taking changes between speaker and listener. Perhaps this causes the patient to feel more involved and therefore more satisfied. Future research should investigate the reasons for large variation of face gaze and the association between face gaze and other outcomes.

Our findings suggest that researchers seeking insight into gaze behaviour could for pragmatic reasons choose to study a short first slice, instead of the whole interaction. Although similar findings were previously reported when using less novel technologies to study gaze^35–37^, it was unclear to date what the optimal duration of this slice should be and whether these findings could be replicated using wearable eye-tracking. Our results suggest that whereas all slice durations predict subsequent face gaze, their predictive value differs. The gain in predictive value from the first 30 s to the first 60 s was bigger than from the first minute to the first 120 s. Therefore, using a 1 min slice of face gaze may provide the right balance between a relatively strong association based on a relatively short slice. The advantage of a larger slice is that a smaller slice likely overestimates the level of face gaze more, since our findings show a small but steady decrease in face gaze over the course of the interaction. Therefore, when choosing to assess face gaze in out-patient consultations based on a thin slice, an onverestimation of the level of face gaze is possible. Another reason to select the first minute of face gaze as a thin slice, is the importance of the first minute—also labelled ‘the golden 60 s’—for the quality of the total interaction between physician and patient^42–44^. There are guidelines for optimizing medical communication that instruct the physician not to interrupt the patient during the first 60 s^45,46^. For other settings, researchers should first investigate which slice duration is the most predictive.

Our study has some limitations. First of all, we studied face gaze in a patient-physician interaction. Our findings may not be generizable to other types of interaction context. Future research should replicate our study in other interactional settings. Furthermore, some of the interactions in our sample were triadic, involving not only a physician and a patient, but additionally a caregiver. The software we used classified the face gaze towards the caregiver as face gaze towards the patient. Therefore, the presence of a caregiver could have overestimated the assessed amount of face gaze^47^. Second, we excluded the eye-tracking data collected during physical examinations. Face gaze could have taken place during physical examinations, possibly decreasing the total amount of face gaze that we assessed. Lastly, even though gaze behaviour is largely unconcious, one can never exclude the possibilty of a Hawthorne effect, meaning that our effects may have been biased because physicians were aware of being observed^48^.

Our study also has strengths. The use of mobile eye-tracking enabled us to study gaze in detail. We have tried to create more understanding of face gaze measures and in real life interactions, thus providing methodological guidance for future research. Furthermore, we included eye-tracking data of 100 real-life interactions—a much higher amount than in previous face-to-face mobile eye-tracking studies, especially considering an applied setting such as medical consultations.

In sum the three measures of face gaze, duration, frequency, and dwell time can all be used to study nonverbal behaviour, but the results they generate may hold different conclusions. All measures can be studied using a thin slice to make inferences about the complete interaction, especially dwell time. Our findings offer guidance in methodological choices for researchers interested in face gaze in dyadic interactions when using wearable eye tracking technologies.

## Methods

### Design

This analysis is part of a prospective, observational study aimed at studying the association of physician face gaze and patient reported outcome measures, primarily trust. The current study focuses on comparing different face gaze measures and investigating face gaze patterns. The study was conducted at the internal medicine outpatient clinic of a Dutch academic hospital. First, sociodemographic characteristics of physicians and patients, and potential confounders (e.g., patients’ social anxiety and attachment style) were queried^49^. Then, the physician wore Tobii Pro eye-tracking glasses to assess face gaze towards patients during the consultation^50^. To register verbal and non-verbal cues (other than face gaze) we used an additional video camera. Questionnaires assessing outcome measures (e.g., satisfaction with the consultation), were queried directly after the consultation. Here we report on the eye-tracking data only. The study was evaluated by the Medical Ethical Committee of the AmsterdamUMC, Location AMC. The Medical Ethical review board qualified our study with the ‘exempt’ status, meaning that review by the full institutional review board was not required. All observations were performed in accordance with relevant guidelines and regulations.

### Participants

Patients and physicians participated in this study during randomly selected check-up outpatient consultations. Patients and physicians were included who had not met prior to their participation in this study. All physicians were residents in internal medicine. We identified check-up outpatient consultations of patients and physicians whom had not previously met, by including physicians who took over patient shifts of other physicians. We selected these consultations to ensure unfamiliarity of patients and physicians and to include relatively homogeneous content of the consultations. The same physicians conducted several consultations, each time with different patients. Eligible patients spoke fluent Dutch, were at least 18 years old and did not have any mental illnesses that could influence study results as judged by the physician.

### Procedure

After physicians agreed to participate, patients were contacted telephonically to ask for participation. Informed consent forms were signed before participating. Physicians and patients responded to a questionnaire assessing socio-demographic characteristics prior to the consultation. Before participating in the study, we assessed whether physicians’ eyes were suitable for a sufficient calibration quality as indicated by the Tobii Pro Glasses Controller software^51^. With the Tobii Pro Glasses 2 calibration is done by having the participants’ gaze focussed on a specific calibration target (black dot on a calibration card) at 0.75–1.25 m distance. The calibration was performed in the consultation room: the physician was seated in his/her chair and the researcher calibrated the physician’s eyes with the calibration card at the location where the patient’s head would later be. The calibration assures the accuracy of the measurement of the eye-tracking glasses and sufficient calibration quality is a prerequisite for collecting precise eye-tracking data ^52^. Lighting conditions were kept constant by conducting all consultations in a room without windows and constant artificial lighting. During the consultation the gaze of the physicians was tracked. When physical examination of the patient was needed, physicians were instructed to take off the eye-tracking glasses. Caregivers of the patients were allowed to be present during the consultation. After the consultation patients responded to the five-item Patient Satisfaction Questionnaire^53^. Responses are provided using Visual Analogue Scales (ranging from 0 to 100), and an example item is “How well did the counsellor address your needs?” The Cronbach’s alpha for our sample was 0.93. Data collection started in February 2018 and ended in May 2019.

### Data analyses

All data without noticeable data loss were included. Because the quality of the calibration was tested beforehand on each physician, all eyes were suitable for eye-tracking measures. Whenever technical issues occurred (e.g., an empty battery of the eye-tracking glasses) data were excluded. The eye-tracking data were analysed with software to automatically create Areas-of-Interest around the faces of all individuals present in the videos, created for this study based on a previously developed algorithm^34^. The output of the software indicated for each video frame (40 ms) whether the gaze of the physician was focused on the face of the patient or the caregiver (if present), i.e., within the Area-of-Interest (coded as ‘1’) or not (‘0’). The software can be downloaded following this link: https://osf.io/4uy35/?view_only=785a011774cf4c4f8c5e4608b34a2a38. For more details on the analyses see Jongerius et al.^34^. The output data, indicating whether the physician was gazing towards the face of the patient, was used as input for the statistical analyses and was manipulated in R Studio^54^. For our R script see: https://osf.io/9y6xq/?view_only=6cd116034ddf4318bbe479bde1612708.

To answer our research questions we calculated three measures of face gaze: (a) face gaze duration per minute; (b) face gaze frequency per minute; and (c) average dwell time, i.e., the average duration per instance of face gaze. Dwell time was operationalized as the duration that the physician gazed uninterrupted at the patient’s face. These three measures formed the basis of our data descriptions and our data analyses.

We performed our analyses three times using the abovementioned face gaze measures, i.e., duration per minute, frequency per minute and average dwell time. To assess how these measures were related, we first examined them descriptively by calculating means and standard deviations, or medians and ranges when the data were not normally distributed. For distributions of the data see histograms in the Supplementary Figs. S1, S2 and S3. We also used histograms of the average duration per minute, average frequency per minute and average dwell time of the face gaze during the interaction subdivided in deciles of time. To test for changes in the amount of face gaze over time, we did time series analyses. We tested whether face gaze duration, frequency and dwell time changed over the course of the interaction. Therefore, we divided face gaze in deciles of time of the consultations. We used linear mixed effect meta-analyses on the aggregated, average values of each decile. To investigate the influence of consultation duration, we additionally calculated Pearson correlations between all three measures of face gaze and the total duration of the consultation and patient satisfaction of the consultation. Furthermore, we performed pairwise Pearson correlation analyses between the face gaze duration, the face gaze frequency and the dwell time. We applied the Bonferroni correction to prevent Type I errors. Therefore, we divided the alpha of 0.05 with 3 meaning that the Pearson correlations were considered significant with an alpha of *p* < 0.017. For our second aim, i.e., to test whether the first brief period of face gaze predicted face gaze during the remainder of the interaction, we performed multilevel analyses using the first 30, 60 and 120 s of face gaze as predictors for face gaze during the remainder of the interaction. Since our data were nested per physician, we extended all linear models with a random effect to compensate for the nested data (per physician). We performed the multilevel analyses three times: for duration, frequency and dwell time of face gaze, each time using total consultation duration as confounding factor. All test outcomes were considered significant with an alpha of *p* < 0.05 or smaller when using the Bonferroni correction and were performed using R v3.6.1 and RStudio 1.2.1335^54^.

## Supplementary Information

Supplementary Information.

## Data Availability

Data where patients are visible cannot be made publicly available. The R script is available at https://osf.io/9y6xq/?view_only=6cd116034ddf4318bbe479bde1612708.
