# Supplementary material for: Eye-tracking analyses of physician face gaze patterns in consultations
Source: Sci Rep. 2021 Oct 6;11:19823. doi: 10.1038/s41598-021-99068-4 (PMC8494763; doi:10.1038/s41598-021-99068-4)
Supplement: Supplementary file 1 — Supplementary Information. [file 41598_2021_99068_MOESM1_ESM.docx]

**Eye-tracking analyses of physician face gaze patterns in consultations**

C. Jongerius, H.G. van den Boorn, T. Callemein, N.T. Boeske,

J. A. Romijn, E. M.A. Smets, M. A. Hillen

**Supplementary Information**

**Supplementary Table S1.** Results of the linear regression mixed-effects models to predict face gaze duration per minute, frequency per minute, and dwell time with a varying slice time of 30, 60 or 120 seconds. For each outcome, we used the level of face gaze in the initial slice, as well as total consultation duration in minutes and a model intercept. We added a random effect for each physician. The model fixed effects parameters are displayed for each linear model.

| **Face gaze outcome** | **Linear regression models** | | | |
| --- | --- | --- | --- | --- |
| Face gaze duration in seconds per minute | Duration of the predicting slice | Intercept | Face gaze duration in seconds in the predicting slice | Total duration of the consultation in minutes |
|  | 30 seconds | 17,42*** | 0,68*** | -0,12 (NS) |
|  | 60 seconds | 11,62** | 0,47*** | -0,12 (NS) |
|  | 120 seconds | 12,44*** | 0,24*** | -0,13 (NS) |
| Face gaze frequency per minute | Duration of the predicting slice | Intercept | Face gaze frequency in the predicting slice | Total duration of the consultation in minutes |
|  | 30 seconds | 19,50*** | 0,53*** | -0,19 (NS) |
|  | 60 seconds | 16,32*** | 0,38*** | -0,18 (NS) |
|  | 120 seconds | 10,86** | 0,26*** | -0,04 (NS) |
| Face gaze dwell time in seconds | Duration of the predicting slice | Intercept | Face gaze dwell time in seconds in the predicting slice | Total duration of the consultation in minutes |
|  | 30 seconds | 1,09*** | 0,31*** | -0,01 (NS) |
|  | 60 seconds | 0,95*** | 0,31*** | -0,01 (NS) |
|  | 120 seconds | 0,72*** | 0,45*** | -0,01 (NS) |

*Note:* NS = not significant, * = p<.05, ** = p<.01, *** = p<.001

**Supplementary Figure S1.** Distribution of physician average face gaze duration per minute

**
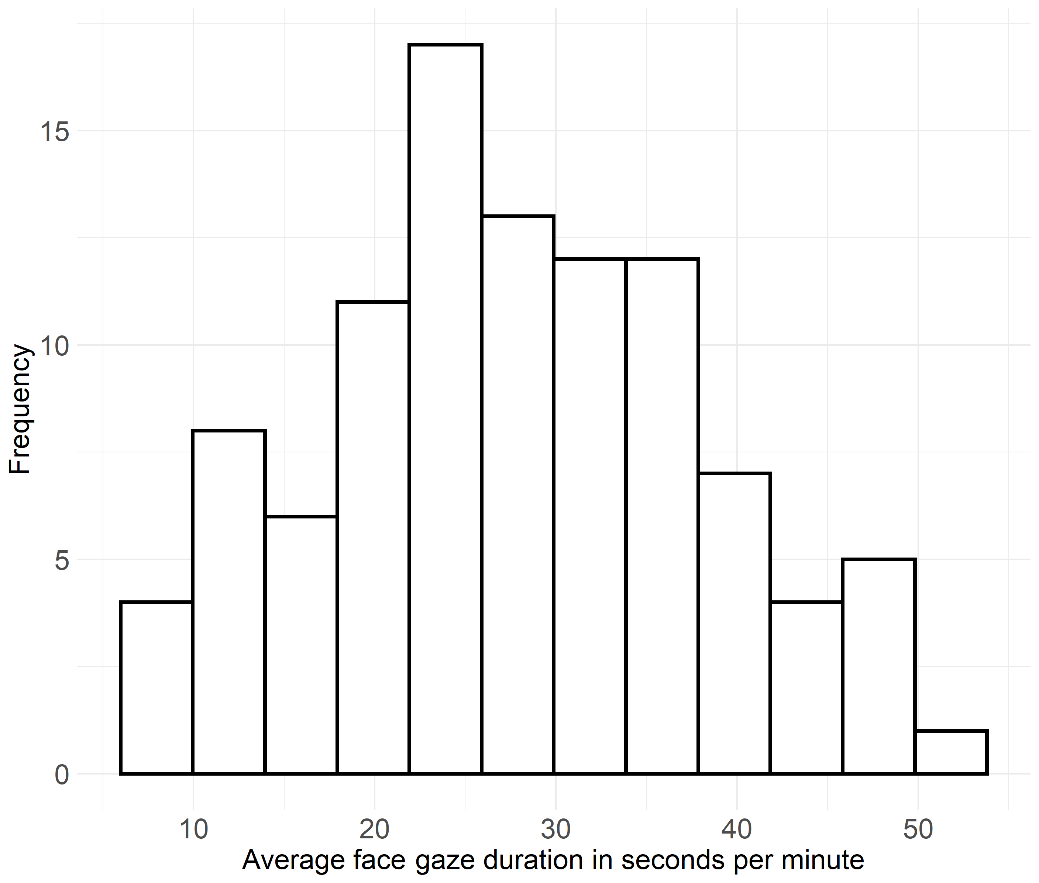
**

**Supplementary Figure S2.** Distribution of physician average face gaze frequency per minute

**
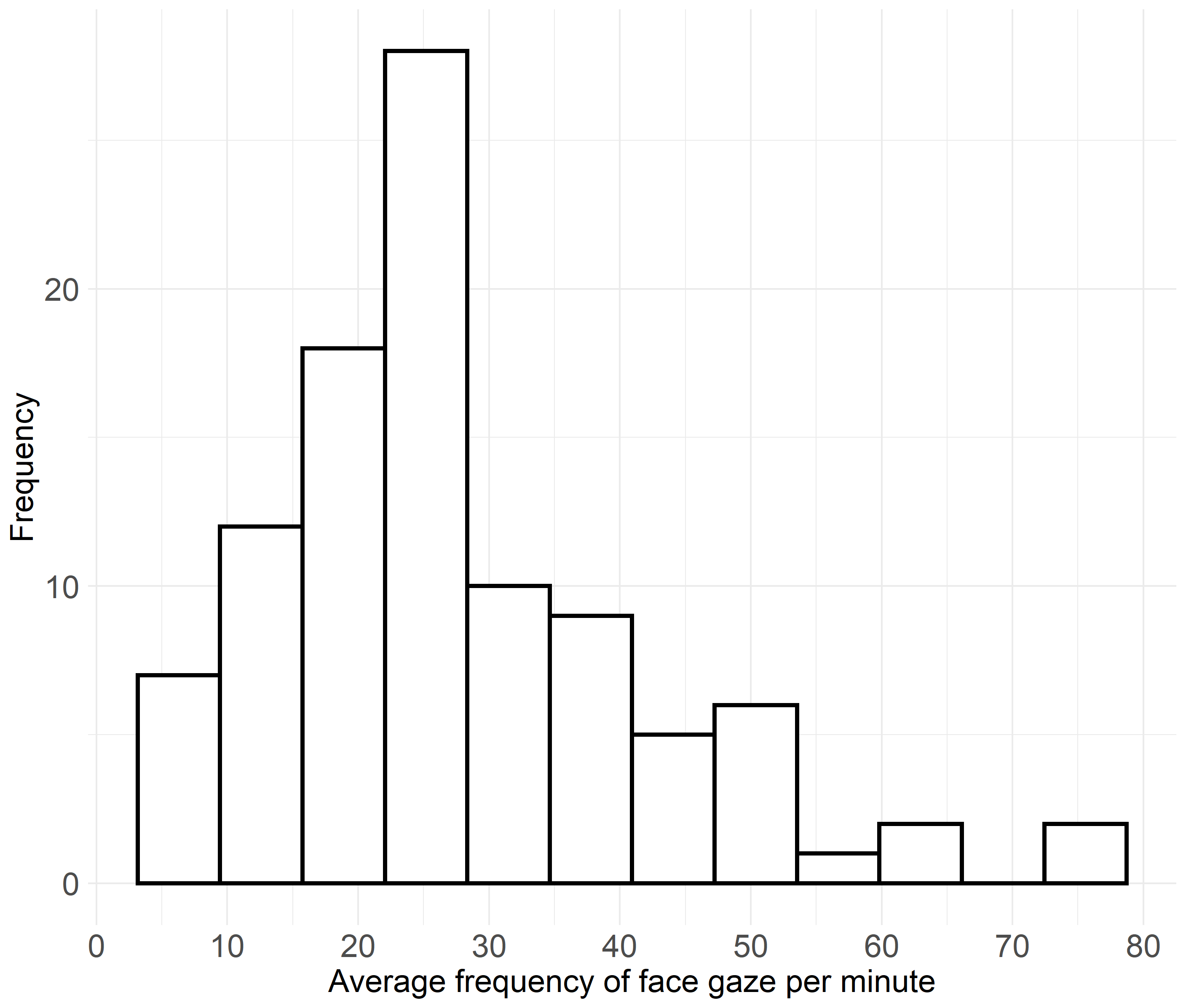
**

**Supplementary Figure S3.** Distribution of physician’s average face gaze dwell time

**
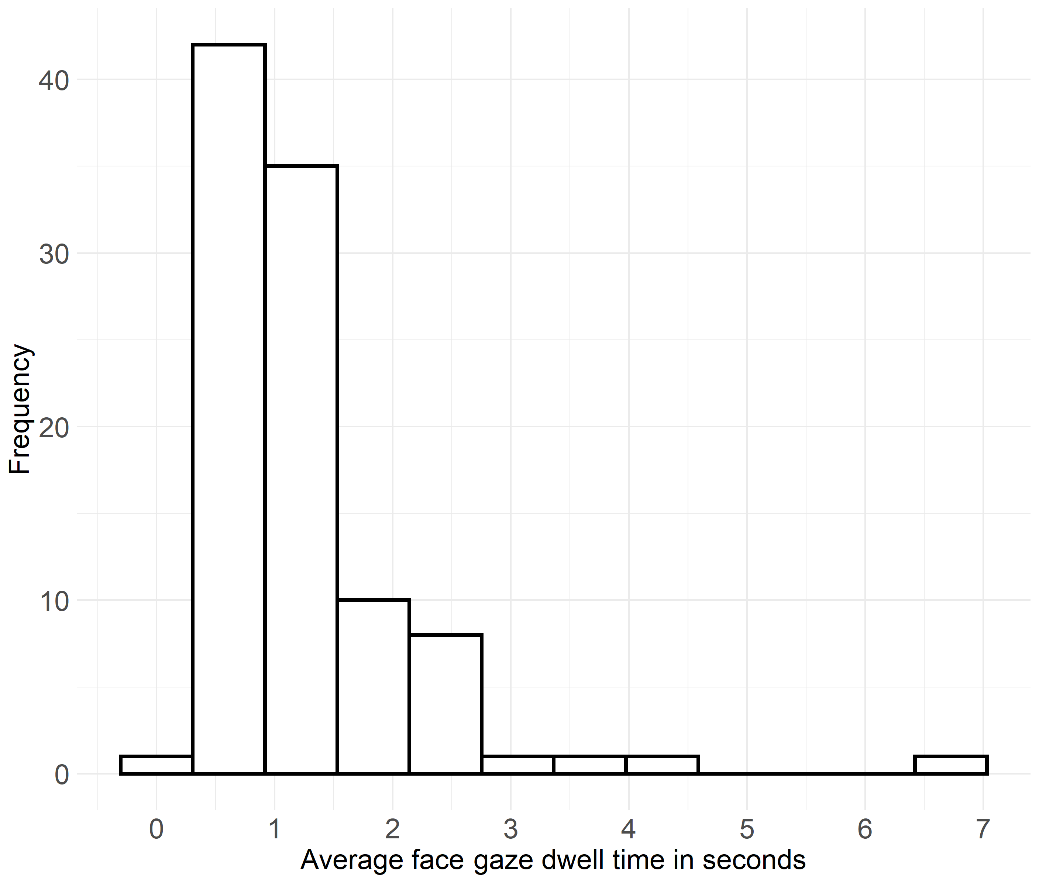
**
